# Supplementary material for: Engineered cell differentiation and sexual reproduction in probiotic and mating yeasts
Source: Nat Commun. 2022 Oct 19;13:6201. doi: 10.1038/s41467-022-33961-y (PMC9582028; doi:10.1038/s41467-022-33961-y)
Supplement: Supplementary file 1 — Supplementary Information [file 41467_2022_33961_MOESM1_ESM.pdf]

Engineered cell differentiation and sexual reproduction in probiotic and mating yeasts

Jensen *et al*.

Table of Contents

Supplementary Fig. 1	1
Supplementary Fig. 2	3
Supplementary Fig. 3	4
Supplementary Fig. 4	5
Supplementary Fig. 5	6
Supplementary Fig. 6	7
Supplementary Table 1	8

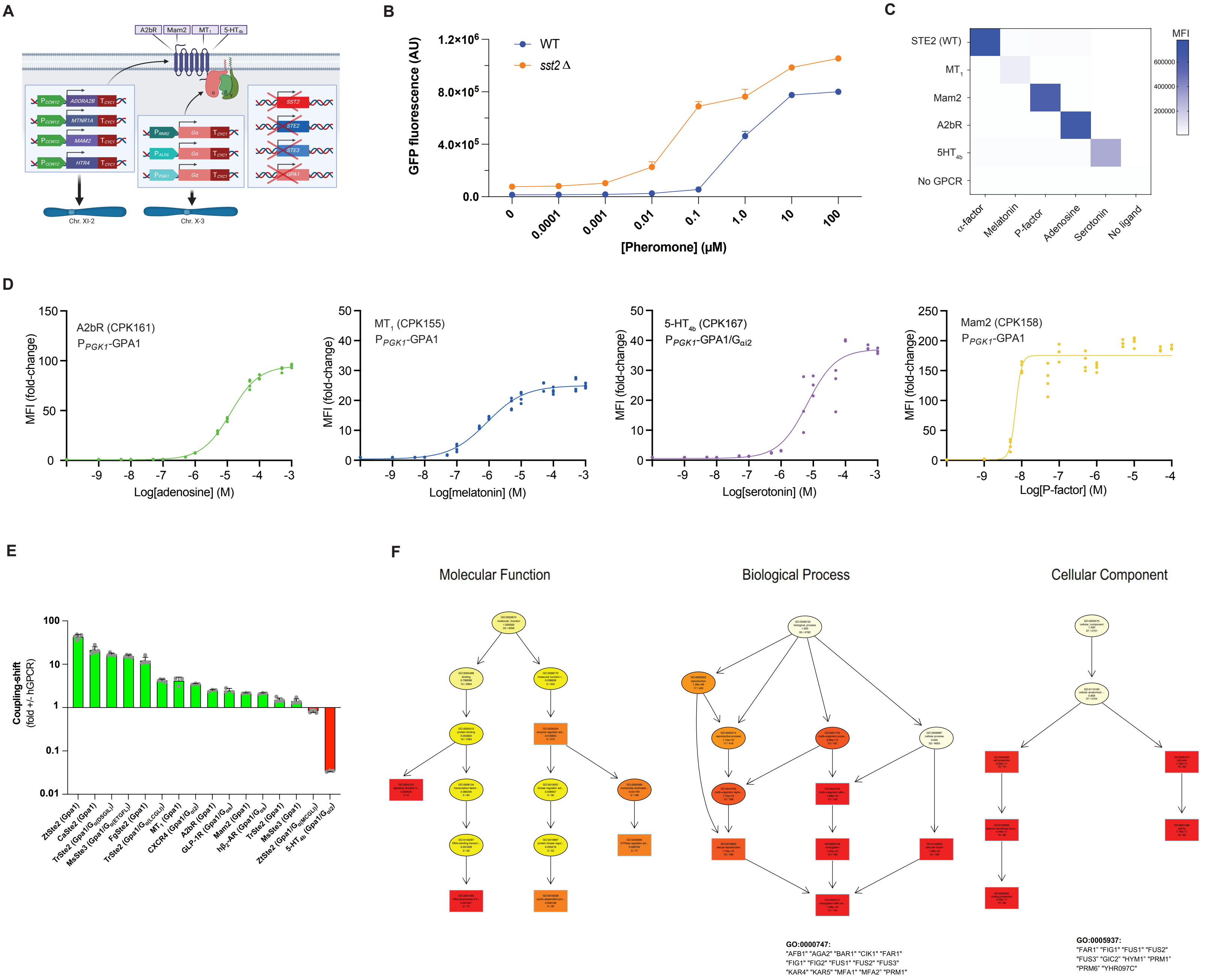

Supplementary Figure 1. (**A**). Schematic overview on engineering of biosensing yeast strains. Heterologous GPCRs (hGPCRs) are overexpressed from the *CCW12* promoter and integrated in the genome, and the *CYC1* terminator attenuated transcription in all designs. Genome-integrated G_α subunits were expressed from the *RNR2*, *ALD6*, or *PGK1* promoters. Four native genes, *SST2*, *STE2*, *STE3,* and *GPA1*, were genetically deleted, and G_β and G_γ subunits are illustrated in a trimeric complex with G_α subunit. (**B**). Native Ste2-signaling in wild-type (CPK86) and sensitized *sst2Δ* (CPK503) yeast strains from increasing concentrations of supplemented pheromone (α-factor). The *FUS1* promoter was used to drive expression of a GFP reporter in response to supplemented pheromone. Data represent means and standard deviations from three biological replicates. AU: artificial units. (**C**). A 6 x 6 orthogonality matrix was made by supplementation of 10 μM of each indicated ligand to individual heterologous GPCRs contained in strains CPK155, CPK158, CPK161, CPK167 and wild-type Ste2 (CPK86). GPCR-signaling

was determined from mean fluorescence intensities (MFI) with a GFP reporter. No GPCR (CPK133) and no ligand controls were included as shown. Data represent means of three biological replicates for all samples. (**D**). High resolution dose-response curves used for calculating EC50 values (*See Suppl. Table 1*). Data represent means and standard deviations from at least three biological replicates. (**E**). Coupling-shifts are presented as log-scaled fold changes in fluorescence from a P_{FUS1}-*GFP* reporter following hGPCR integration (+hGPCR: strains SBY143, SBY146, CPK153, CPK156, CPK159, CPK165, and CPK450-459) over background (no hGPCR: SBY123, CPK131, CPK134, CPK343, CPK347, CPK350, and CPK424). Specific G_α subunits are indicated for each hGPCR presented in the plot. Means and standard deviations represent at least three biological replicates. (**F**). The GO trees induced by the top 5 GO terms for "molecular function", "biological process", and "cellular component" respectively. Rectangles indicate the 5 most significant terms. The color represents the relative significance, ranging from dark red (most significant) to bright yellow (least significant). For each node, the first two lines show the GO information. The third line is the raw p-value, and the fourth line shows the number of significant genes and the total number of genes annotated to the respective GO term.

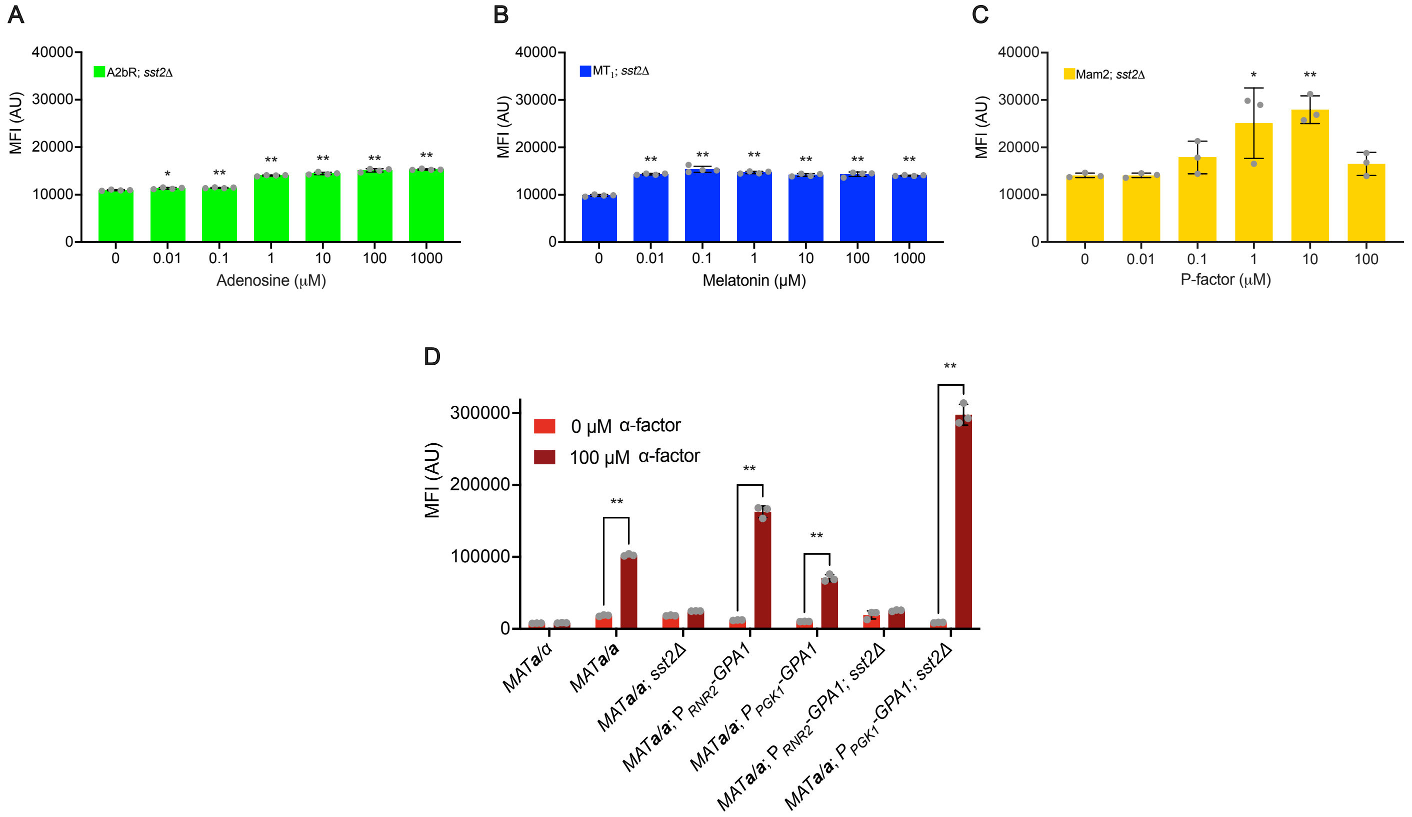

Supplementary Figure 2. (**A-C**). Median fluorescence intensity (MFI) shown in artificial units (AU) from plasmid-based P_{FUS1}-*GFP* reporter expression in *S. boulardii sst2Δ* biosensing strains for (**A**). A2bR sensing adenosine (SB45), (**B**). MT_1 sensing melatonin (SB46), and (**C**). Mam2 sensing P-factor (SB47). (**D**). Mating pathway stimulation with α-factor in engineered *S. boulardii* strains: SB14 (*MAT***a**/α), SB17 (*MAT***a**/**a**), SB36 (*MAT***a**/**a**; *sst2Δ*), SB40 (*MAT***a**/**a**; P_{RNR2}-*GPA1*), SB39 (*MAT***a**/**a**; P_{PGK1}-*GPA1*), SB38 (*MAT***a**/**a**; P_{RNR2}-*GPA1*; *sst2Δ*), and SB37 (*MAT***a**/**a**; P_{PGK1}-*GPA1*; *sst2Δ*). Means and standard deviations represent at least three biological replicates. Statistical significance was determined relative to no ligand supplementation (0 μM) controls using two-way analysis of variance (ANOVA) using GraphPad Prism (*$p > 0.05$, **$p \leq 0.01$).

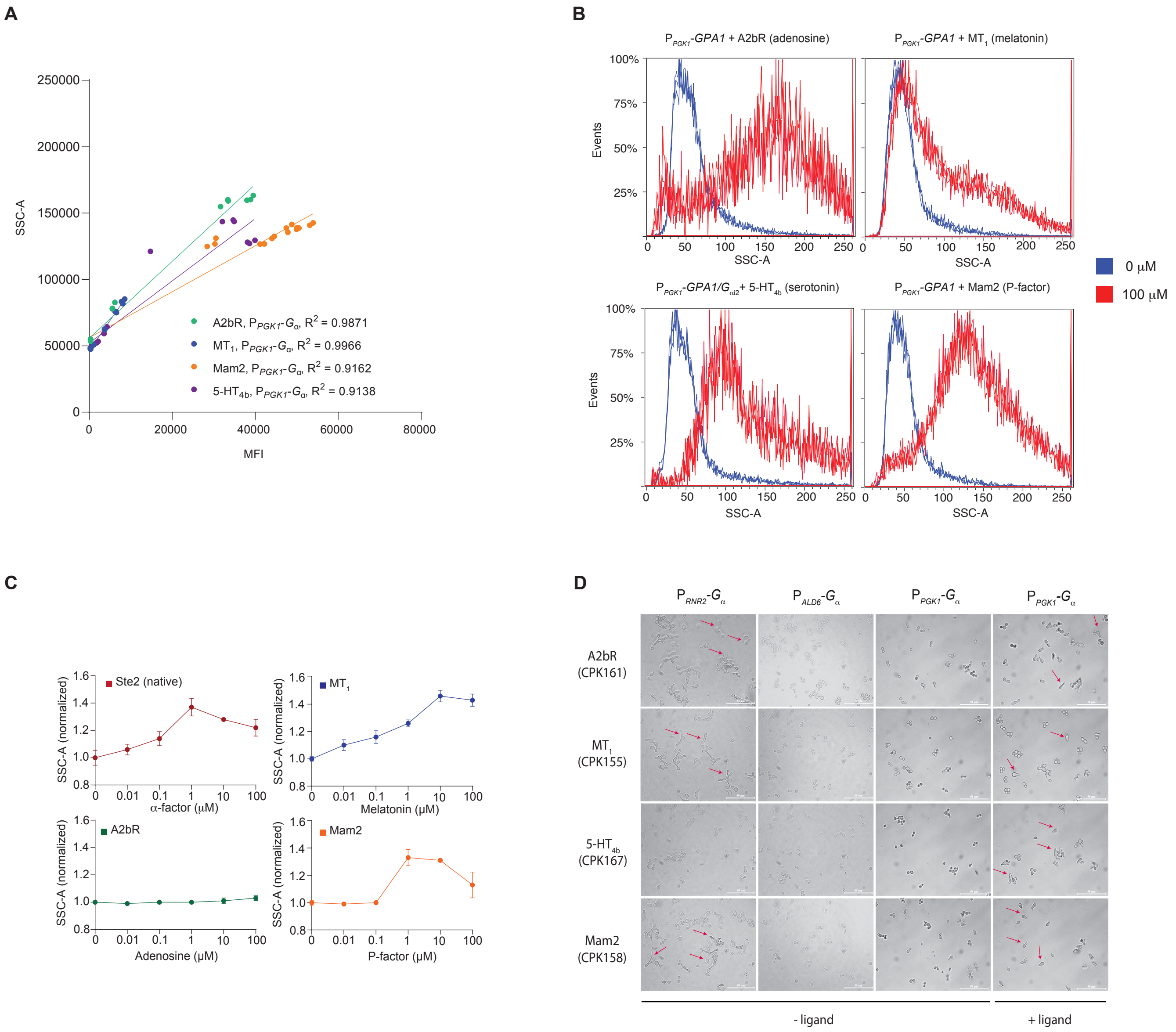

Supplementary Figure 3. (**A**). Side-scatter (SSC-A) as a linear function of median fluorescence intensity (MFI) for strains CPK155, CPK158, CPK161, and CPK167. (**B**). Histograms showing increased SSC-A during hGPCR-signaling in strains CPK155, CPK158, CPK161, and CPK167 from incubation with cognate ligands (100 µM, red) as compared to no hGPCR-signaling (0 µM, blue). (**C**). SSC-A changes with cognate ligand concentration in biosensing *S. boulardii* (SB17 and SB48-50). SSC-A is normalized internally for each biosensing strain to no ligand supplementation controls (0 µM). Data represent means and standard deviations from three biological replicates. (**D**) Representative shmoo morphologies (red arrows) in S. cerevisiae strains in absence (-ligand) or presence of cognate ligands (+ligand, 100 µM) for A2bR, MT1, and 5-HT4b, or Mam2 (1 µM). The experiment was repeated more than three times. Size bars illustrate 50 µm.

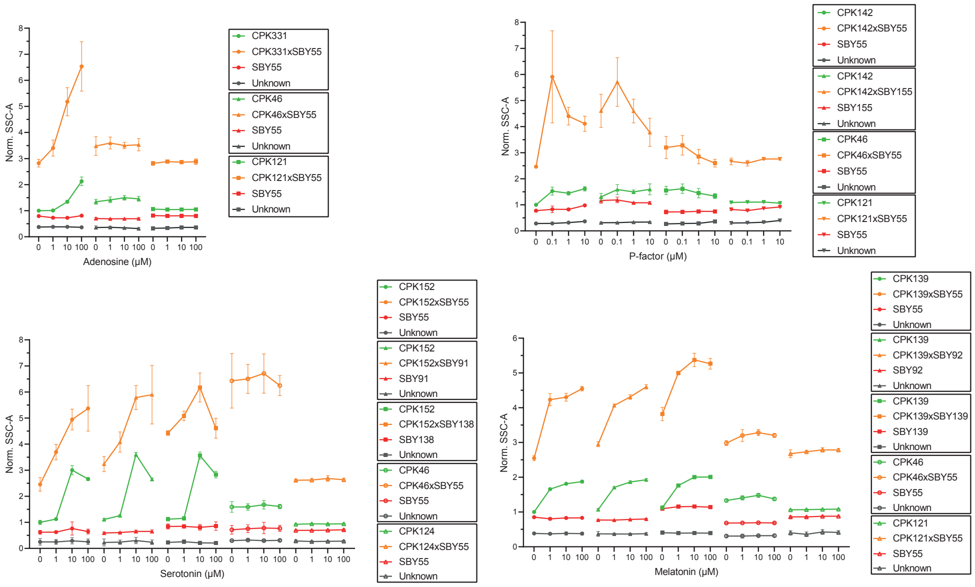

Supplementary Figure 4. Alterations in cellular morphology during synthetic mating in the formation of diploids (CPKxSBY) and shmooing haploids (CPK and SBY), interpreted by side-scatter area (SSC-A). SSC-A was measured simultaneously with mating data acquisition. Cells are identified by flow cytometry. SSC-A is normalized to the examined CPK-strain without supplementation or production of ligand. CPK46xSBY55 and CPK121xSBY55 or CPK124xSBY55 were used as references for positive and negative mating pair controls with relevant ligand supplementation, respectively. Results are presented as means with standard deviations determined from five biological replicates. Normalized SSC-A for semi-synthetic mating trials for adenosine in Fig. 4B, P-factor in Fig. 4C, melatonin in Fig. 4D, and serotonin in Fig. 4E.

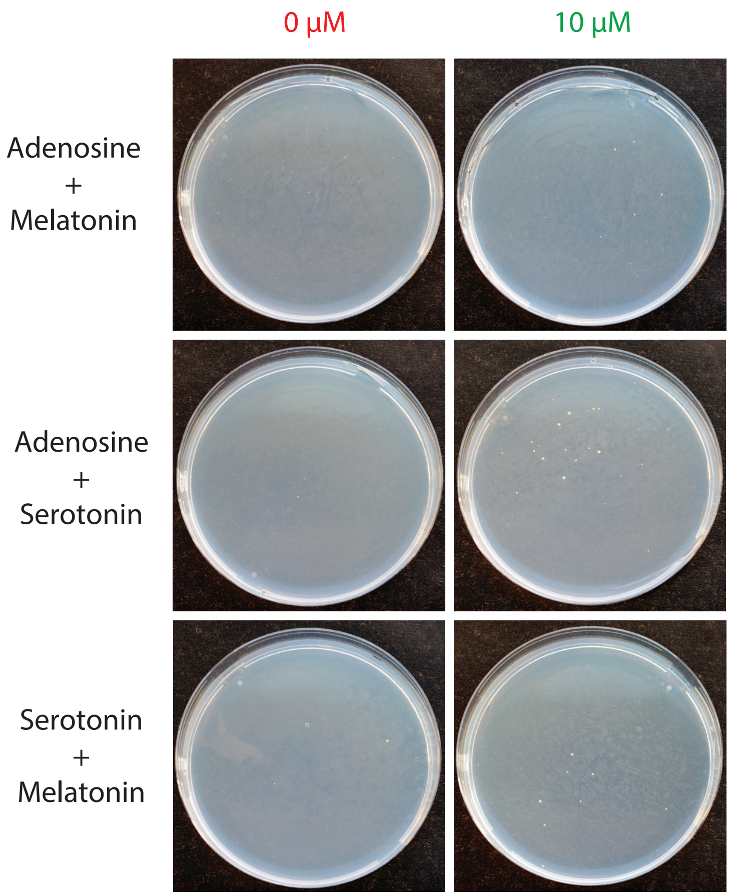

Supplementary Figure 5. Representative pictures of plated full synthetic mating trials for supplementation (10 μM) of both ligands in combination, or no supplementation (0 μM), for crosses with strains corresponding to CPK139xSBY157 (adenosine+melatonin), CPK152xSBY157 (adenosine+serotonin), and CPK152xSBY156 (melatonin+serotonin). CPK139 and CPK152 were transformed with plasmid pEDJ400 (*URA3*) before these trials to allow diploid selection on SC-UW. 95 μl of co-culture was plated on each SC-UW plate, and pictures were taken following incubation at 30 °C for 7 days.

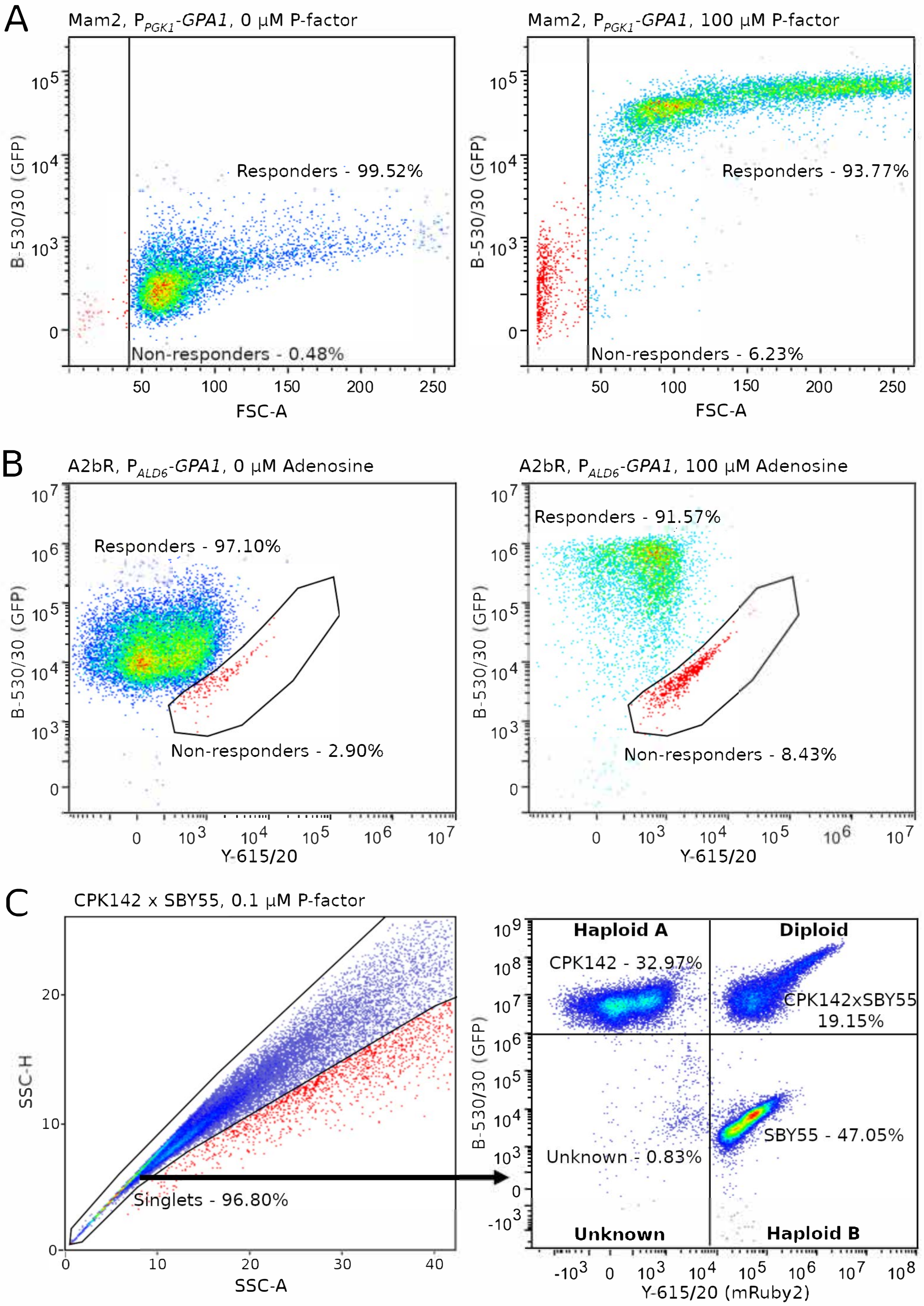

Supplementary Figure 6. Flow cytometry data gating strategy. **A**. Representative image of general removal of small non-responsive cells in GFP-based biosensor assays by an exclusive gate on the FSC-A axis, shown for both an unactivated (left) and activated (right) state. **B**. Representative image of removal of non-responsive cells in GFP-based biosensor assays based on distinct autofluorescence seen when plotting cells in green (B-530/30) against red (Y-615/20) dimensions, shown for both an unactivated (left) and activated (right) state. **C**. Representative image of general gating for singlets (SSC-A vs SSC-H) and for quadrant gating applied in the double-fluorescence dimension (Y-615/20 vs B-530/30) for mating assays, to classify cells as haploid A, haploid B, or diploid. Alternatively as unknown cells below fluorescence thresholds.

Supplementary Table 1

Saccharomyces cerevisiae			**DrugBank Entries** (https://go.drugbank.com - 04-07-2022)		
**Receptor**	**Gα-subunit**	**Determined**	**EC50 (μM)**	**Mammalian host**	**Source (PubMed ID)**
Ste2 (WT)	native	0.8209	-	-	-
Ste2 (*sst2Δ*)	native	0.0716	-	-	-
A2bR	PPGK1-GPA1	12.73	24	Chinese hamster ovary cells	20541935, 21388809
MT1 (*MTNR1A*)	PPGK1-GPA1	0.8704	2.6E-5, 0.0015, 0.00192, 0.0022, 0.00224	Chinese hamster ovary cells	21568291, 21420861, 12061881, 20444610, 12646022
5-HT4b	PPGK1-GPA1/Gαi2	6.533	0.097 uM	Chinese hamster ovary cells	35452231
Mam2	PPGK1-GPA1	0.006945	-	-	-

Saccharomyces boulardii		
**Receptor**	**Gα-subunit**	**Determined**
Ste2, MAT**a**/α	native	-
Ste2, MAT**a**/**a**	native	0.3349
MT1	PPGK1-GPA1	0.02819
A2bR	PPGK1-GPA1	0.9454
Mam2	PPGK1-GPA1	5.273
