# Supplementary material for: Engineered cell differentiation and sexual reproduction in probiotic and mating yeasts
Source: Nat Commun. 2022 Oct 19;13:6201. doi: 10.1038/s41467-022-33961-y (PMC9582028; doi:10.1038/s41467-022-33961-y)
Supplement: Supplementary file 2 — Reporting Summary [file 41467_2022_33961_MOESM2_ESM.pdf]

nature research

Corresponding author(s): Michael Krogh Jensen & Emil Damgaard Jensen

Last updated by author(s): Sep 28, 2022

Reporting Summary

Nature Research wishes to improve the reproducibility of the work that we publish. This form provides structure for consistency and transparency in reporting. For further information on Nature Research policies, seeAuthors & Referees and theEditorial Policy Checklist .

Statistics

For all statistical analyses, confirm that the following items are present in the figure legend, table legend, main text, or Methods section.

n/a	Confirmed	
☐	☒	The exact sample size (*n*) for each experimental group/condition, given as a discrete number and unit of measurement
☐	☒	A statement on whether measurements were taken from distinct samples or whether the same sample was measured repeatedly
☐	☒	The statistical test(s) used AND whether they are one- or two-sided<br>*Only common tests should be described solely by name; describe more complex techniques in the Methods section.*
☐	☒	A description of all covariates tested
☐	☒	A description of any assumptions or corrections, such as tests of normality and adjustment for multiple comparisons
☐	☒	A full description of the statistical parameters including central tendency (e.g. means) or other basic estimates (e.g. regression coefficient) AND variation (e.g. standard deviation) or associated estimates of uncertainty (e.g. confidence intervals)
☐	☒	For null hypothesis testing, the test statistic (e.g. *F*, *t*, *r*) with confidence intervals, effect sizes, degrees of freedom and *P* value noted<br>*Give P values as exact values whenever suitable.*
☒	☐	For Bayesian analysis, information on the choice of priors and Markov chain Monte Carlo settings
☒	☐	For hierarchical and complex designs, identification of the appropriate level for tests and full reporting of outcomes
☐	☒	Estimates of effect sizes (e.g. Cohen's *d*, Pearson's *r*), indicating how they were calculated

Our web collection on statistics for biologists contains articles on many of the points above.

Software and code

Policy information about availability of computer code

Data collection	NovoExpress Software (Agilent) v1.6.0<br>BD FACSDiva Software (BD Biosciences) v6.1.3
Data analysis	GraphPad Prism v9.2.0<br>RStudio v1.4.1106 for R v4.1.2<br>Chromeleon™ Chromatography Data System (CDS) Software v7.3.1<br>FlowLogic™ v8.3 (Inivai Technologies)<br>Trimmomatic v0.39<br>STAR v2.7.9a<br>HTSeq-count v0.13.5<br>edgeR v3.43.1<br>pheatmap v1.0.12<br>ggplot2 v3.3.5<br>TopGO v2.44.0<br>IDT Codon Optimization tool

For manuscripts utilizing custom algorithms or software that are central to the research but not yet described in published literature, software must be made available to editors/reviewers. We strongly encourage code deposition in a community repository (e.g. GitHub). See the Nature Research guidelines for submitting code & software for further information.

Data

Policy information about availability of data

All manuscripts must include a data availability statement. This statement should provide the following information, where applicable:

- Accession codes, unique identifiers, or web links for publicly available datasets
- A list of figures that have associated raw data
- A description of any restrictions on data availability

All raw sequencing data used for transcriptome analysis were deposited into the NCBI Short Read Archive under the accession number (PRJNA790752).

Figure list:
- Figure 1 B-D
- Figure 2 B-E
- Figure 3 A-C & E
- Figure 4 B-E
- Figure 5 B-G
- Suppl. Fig. 1C-E
- Suppl. Fig. 2A-D
- Suppl. Fig. 3A-C
- Suppl. Fig. 4

There are no restrictions on data availability when the study is published.

Databases accession links: Unique identifiers are listed in the relevant supplementary tables.
- https://go.drugbank.com
- https://www.uniprot.org
- https://www.ncbi.nlm.nih.gov/sra

Field-specific reporting

Please select the one below that is the best fit for your research. If you are not sure, read the appropriate sections before making your selection.

[x] Life sciences [ ] Behavioural & social sciences [ ] Ecological, evolutionary & environmental sciences

For a reference copy of the document with all sections, see nature.com/documents/nr-reporting-summary-flat.pdf

Life sciences study design

All studies must disclose on these points even when the disclosure is negative.

Sample size	We used 4 different conditions for 5 different strains in 3 replicates for transcriptome analysis. We used ≥ 3 biological replicates for all flow cytometry and mating analyses. Results were highly reproducible between replicates, thus making the chosen samples sizes sufficient for conducted analyses.
Data exclusions	Exclusion gating of non-responsive subpopulations during GPCR characterization (see gating strategy below).
Replication	n ≥ 3 successful attempts.
Randomization	Colonies were randomly picked from transformation plates.
Blinding	Beyond randomization of colony picking no blinding was needed in this study, because the work involves rationally engineered strains.

Reporting for specific materials, systems and methods

We require information from authors about some types of materials, experimental systems and methods used in many studies. Here, indicate whether each material, system or method listed is relevant to your study. If you are not sure if a list item applies to your research, read the appropriate section before selecting a response.

Materials & experimental systems

n/a	Involved in the study
☒	☐ Antibodies
☒	☐ Eukaryotic cell lines
☒	☐ Palaeontology
☒	☐ Animals and other organisms
☒	☐ Human research participants
☒	☐ Clinical data

Methods

n/a	Involved in the study
☒	☐ ChIP-seq
☐	☒ Flow cytometry
☒	☐ MRI-based neuroimaging

Flow Cytometry

Plots

Confirm that:

☐ The axis labels state the marker and fluorochrome used (e.g. CD4-FITC).

☒ The axis scales are clearly visible. Include numbers along axes only for bottom left plot of group (a 'group' is an analysis of identical markers).

☐ All plots are contour plots with outliers or pseudocolor plots.

☒ A numerical value for number of cells or percentage (with statistics) is provided.

Methodology

Sample preparation	Single colonies consisting of yeast cells were inoculated and grown O/N, then diluted 10-fold and allowed for another O/N incubation, and on day 3 diluted to OD = 0.2 and incubated for 2 hrs prior to flow cytometry with ligand incubation running for 4 hrs, or used without additional incubation for mating trials running for 5 hrs.
Instrument	BD LSRFortessa™ X-20 (BD Biosciences) and NovoCyte Quanteon™ (Agilent)
Software	FlowLogic™ v8.3 (Inivai Technologies)
Cell population abundance	For NovoCyte Quanteon measurements FSC-H >150,000 was used as threshold. For BD LSRFortessa FSC = 240V and SSC = 267V were used.
Gating strategy	All flow cytometry data was extracted as FCS files and gated in FlowLogic™ v8.3 (Inivai Technologies). SSC-A, FSC-A, and fluorescence data points were derived from median values and median fluorescence intensities (MFI) of gated populations. Normalized MFI (nMFI) was obtained by normalizing to the mean of background MFIs. For statistics and data analysis means of medians and MFIs were applied ± standard deviation. Population proportions were derived directly from event counts per gate, including diploid frequencies in mating trials. For PFUS1-GFP-based hGPCR characterization and biosensor assays, small non-responsive cells were removed by consistent exclusive gates based on a minimum FSC-A values. In the case of normal-sized, but non-responsive, cells, their distinct autofluorescence was used to create an exclusive gate (B-530/30 vs Y-615/20). For mating trials, firstly, compensation was done between Y-615/20 and B-530/30 using CompLogic automatic compensation in FlowLogic™ v8.3 (Inivai Technologies). Secondly, singlets were gated based on SSC (SSC-A vs SSC-H). Lastly, a quadrant gate was applied in the double-fluorescence dimension (Y-615/20 vs B-530/30) to classify all detected cells as either identifiable haploid A, haploid B, or diploid, or alternatively unidentifiable unknown cells lying below fluorescence thresholds. The quadrant gate was established by employing fluorescence minus one (FMO) controls.

☒ Tick this box to confirm that a figure exemplifying the gating strategy is provided in the Supplementary Information.
