# Supplementary material for: Engineered cell differentiation and sexual reproduction in probiotic and mating yeasts
Source: Nat Commun. 2022 Oct 19;13:6201. doi: 10.1038/s41467-022-33961-y (PMC9582028; doi:10.1038/s41467-022-33961-y)
Supplement: Supplementary file 4 — Description of Additional Supplementary Files [file 41467_2022_33961_MOESM4_ESM.pdf]

Title: Supplementary Data 1.
Description: Reads per kilobase of transcript per million reads mapped (RPKM) values for all differentially expressed genes (DEGs) from transcriptome data analysis.

Title: Supplementary Data 2.
Description: Transcriptome analysis subset showing reads per kilobase of transcript per million reads mapped (RPKM) values for Gα subunit expression.

Title: Supplementary Data 3.
Description: HPLC analysis showing serotonin and melatonin quantification.

Title: Supplementary Data 4.
Description: Strains used in this study.

Title: Supplementary Data 5.
Description: Plasmids used in this study.

Title: Supplementary Data 6.
Description: Oligos used in this study.

Title: Supplementary Data 7.
Description: Gene blocks used in this study.

Title: Supplementary Data 8.
Description: GPCRs used in this study.

Title: Supplementary Data 9.
Description: Curve fit and ANOVA analyses for Figure 1B and Supplementary Figures 1B & 1D.

Title: Supplementary Data 10.
Description: Curve fit and ANOVA analyses for Figure 2 and Supplementary Figure 2.

Title: Supplementary Data 11.
Description: Pearson correlation plot and regression analyses for Figure 3 and Supplementary Figure 3.

Title: Supplementary Data 12.
Description: ANOVA analyses for Figure 4.

Title: Supplementary Data 13.
Description: ANOVA analyses for Figure 5B-F.

Title: Supplementary Data 14.
Description: ANOVA analyses for Figure 5G.
